# Prognostic value of cell-free DNA in cerebrospinal fluid from lung cancer patients with brain metastases during radiotherapy

**DOI:** 10.1186/s13014-023-02239-y

**Published:** 2023-03-11

**Authors:** Simiao Qiao, Yuying Hao, Linbo Cai, Xiaotong Duan, Lijuan Wang, Aidong Zhou, Xiaoxia Zhu

**Affiliations:** 1 Department of Radiation Oncology, Zhujiang Hospital, Southern Medical University, 253 Industrial Avenue, Guangzhou, 510282 China; 2 Department of Oncology, Guangdong Sanjiu Brain Hospital, Guangzhou, 510515 China

**Keywords:** NSCLC, Brain metastases, Radiotherapy, Cell-free DNA, cTMB

## Abstract

**Background:**

During the last decades, radiotherapy (RT) for non-small cell lung cancer (NSCLC) with brain metastases (BM) has been developed. However, the lack of predictive biomarkers for therapeutic responses has limited the precision treatment in NSCLC-BM.

**Patients and methods:**

In order to find the predictive biomarkers for RT, we investigated the influence of RT on the cell-free DNA (cfDNA) from cerebrospinal fluid (CSF) and the frequency of T cell subsets of NSCLC patients with BM. A total of 19 patients diagnosed as NSCLC with BM were enrolled. The CSF from 19 patients and matched plasma samples from 11 patients were collected before RT, during RT, and after RT. The cfDNA from CSF and plasma were extracted, and the cerebrospinal fluid tumor mutation burden (cTMB) was calculated after through next-generation sequencing. The frequency of T cell subsets in peripheral blood was using flow cytometry.

**Results:**

The detection rate of cfDNA was higher in CSF compared to plasma in the matched samples. The mutation abundance of cfDNA in CSF was decreased after RT. However, no significant difference was observed in cTMB before and after RT. Although the median intracranial progression-free survival (iPFS) has not yet been reached in patients with decreased or undetectable cTMB, there was a trend that these patients possessed longer iPFS compared to those with stable or increased cTMB (HR 0.28, 95% CI 0.07–1.18, *P* = 0.067). The proportion of CD4^+^T cells in peripheral blood was decreased after RT. Conclusion: Our study indicates that cTMB can serve as a prognostic biomarker in NSCLC patients with BMs.

**Supplementary Information:**

The online version contains supplementary material available at 10.1186/s13014-023-02239-y.

## Introduction

Brain metastases (BM) are common and lethal for advanced non-small cell lung carcinoma (NSCLC) [1]. About 10% of BM occurs in newly diagnosed NSCLC patients, while 25–40% of them occur after curative treatment [2]. Although therapeutics, including targeted therapy, immunotherapy, and radiotherapy (RT), were rapidly developed in the past decade, the clinical outcomes and quality of life of NSCLC with BM are relatively poor. There is a lack of evidence-based management of NSCLC patients with BMs. Recently, with intensive studies on cancer driver genes and the emergence of novel targeted therapies, it has been proved that some targeted drugs can pass through the blood–brain barrier, which subsequently prevents or control brain metastases for a while [3–6]. However, the treatment efficacy is limited to asymptomatic or stable BM [7], and some patients will develop central nervous system progression eventually [8–10].

Radiotherapy (RT) is the standard treatment for BMs of NSCLC. For patients with driver gene mutation, a combination of targeted therapy with RT might shed light on those patients. However, the conclusions of previously published retrospective clinical studies are inconsistent [11–13]. Currently, the evaluation of treatment efficacy is mainly based on radiographic images, which can neither reflect the treatment efficacy immediately nor the influence of RT on molecular clones of BM, failing to instruct clinicians to design personalized treatment plans. Therefore, biomarkers are urgently needed to predict the efficacy of combination therapy.

For patients without driver gene mutation or those resistant to multiple targeted therapies, immune checkpoint inhibitors (ICIs) provide another promising treatment approach by reinvigorating the immune cells [14]. It has been confirmed that the combination of ICIs and RT can mutually enhance the efficacy in treating BM from NSCLC [15]. However, it is still unclear whether RT can induce and stimulate the immune system of NSCLC patients with BM.

Recently, it has been reported that cell-free DNA (cfDNA) from cerebrospinal fluid (CSF) is more sensitive to the detection of mutated genes than that in plasma, which thus can reflect the treatment response in time [16–19]. In addition, tumor mutation burden (TMB) can be calculated accurately based on large next-generation sequencing (NGS) panel covering a genome area > 1 Mb [20]. In the present study, we explored the dynamic changes of cfDNA in CSF and the frequency of T cell subsets in peripheral blood during RT from NSCLC-BM patients. We figured out for the first time that the change of cerebrospinal fluid tumor mutation burden (cTMB) before and after radiotherapy could predict intracranial progression-free survival (iPFS) in NSCLC patients with BMs.

## Methods

### Patients and sample collection

Nineteen patients were included in the study. All patients were diagnosed as NSCLC with BMs between March 2018 and January 2020 from Southern Medical University or Guangdong Sanjiu Brain Hospital, and the last follow-up time was April 19th, 2020. All patients received chest computed tomography (CT) or positron emission tomography/computed tomography (PET/CT), gadolinium-enhanced magnetic resonance imaging (MRI) of the brain, and pathological examination or CSF cytologic examination or mutation detection via NGS of cfDNA in CSF to confirm the diagnosis of NSCLC with brain or meningeal metastases. Approximately 10 mL CSF was collected by lumbar puncture in each patient before RT (Before RT), in the middle (During RT), and at the end of RT (After RT), and an additional 8 mL of peripheral blood was collected at the same time from 8 patients. Plasma was extracted within 2 h of blood collection, and all samples were shipped to the central testing laboratory within 48 h (Additional file 1: Figure S1). All patients provided signed informed consent, and the study protocol was approved by the research ethics committee of each hospital.

### Targeted NGS and data processing

Circulating cfDNA from plasma was extracted using the QIAamp Circulating Nucleic Acid kit (Qiagen). Sequencing libraries were prepared using the KAPA Hyper Prep Kit (KAPA Biosystems) according to the manufacturer's instructions. Customized xGen lockdown probes (Integrated DNA Technologies) targeting 520 or 474 cancer-relevant genes were used for hybridization enrichment. Then, the capture reaction was performed with Dynabeads M-270 (Life Technologies) and xGen Lockdown hybridization and wash kit (Integrated DNA Technologies), following the manufacturers' instructions. Captured libraries were on-beads PCR amplified with Illumina p5 (5′ AAT GAT ACG GCG ACC ACC GA 3′) and p7 primers (5′ CAA GCA GAA GAC GGC ATA CGA GAT 3′) in KAPA HiFi HotStart ReadyMix (KAPA Biosystems), followed by purification using Agencourt AMPure XP beads. Libraries were quantified by qPCR using the KAPA Library Quantification kit (KAPA Biosystems). Library fragment size was determined by Bioanalyzer 2100 (Agilent Technologies). According to the manufacturer's instruction, the target-enriched library was then sequenced on the HiSeq4000 NGS platform (Illumina). The mean coverage depth was 143× for the whole blood control samples and 4000× for cfDNA samples.

### Sequence alignment and processing

Base calling was performed on bcl2fastq v2.16.0.10 (Illumina, Inc.) to generate sequence reads in FASTQ format (Illumina 1.8 + encoding). Quality control (QC) was performed with Trimmomatic. High-quality reads were mapped to the human genome (hg19, GRCh37 Genome Reference Consortium Human Reference 37) using the BWA aligner 0.7.12 with the BWA-MEM algorithm and default parameters to create SAM files. Picard 1.119 was used to convert SAM files to compressed BAM files, sorted according to chromosome coordinates. The Genome Analysis Toolkit (GATK, version 3.4–0) was used to locally realign the BAMs files at intervals with indel mismatches and recalibrate base quality scores of reads in BAM files.

### Single nucleotide variants (SNV)/short insertions/deletions (InDels)/Copy number variations (CNVs) detections

SNVs and InDels were identified by VarScan2 2.3.9 with minimum variant allele frequency threshold set at 0.01 and *P*-value threshold for calling variants set at 0.05 to generate Variant Call Format (VCF) files. All SNVs/InDels were annotated with ANNOVAR, and each SNV/InDel was manually checked on the Integrative Genomics Viewer (IGV). CNVs were detected using in-house-developed software.

### Statistical analysis

Comparisons of TMB and subset of T cells before RT, during RT, and after RT were done using one-way "repeated measures" ANOVA analysis with a Bonferroni post-hoc test by GraphPad prism 8.0. Comparisons of mutation allele frequency between groups were done using unpaired T-test by GraphPad prism 8.0. Kaplan–Meier curves were compared using the log-rank test for survival analyses, and the Cox proportional hazards model calculated hazard ratios (HR). A two-sided *P* value of less than 0.05 was considered significant for all tests unless indicated otherwise. The rest statistical analyses was done in R (v.3.3.2).

## Results

### Patient characteristics

Between March 2018 and January 2020, 19 patients were screened for enrolment at Southern Medical University or Guangdong Sanjiu Brain Hospital. All patients were diagnosed with NSCLC with pathological examination, except for D04, who was diagnosed by detecting epidermal growth factor receptor (EGFR) 19del in CSF cfDNA. Baseline demographics and clinical characteristics are listed in Table 1 and Additional file 2: Table S1. We detected cfDNA in all 19 patients. In addition, five patients (D06, D07, S01, S03, S08) were diagnosed with leptomeningeal metastasis by CSF cytologic examination. Leptomeningeal metastasis was also found in 3 patients (S02, S05, S07) by neurological symptoms and gadolinium-enhanced MRI. Notably, four patients (D01, D04, D07, S11) did not receive any concurrent system therapy during RT.

**Table 1:** Clinicopathological feature of NSCLC patients with brain metastases and changes of CSF mutation status before and after brain radiotherapy

Patients	Gender	Pathology	PS	GPA	Baseline mutation status at first diagnosed (Sample type)	Radiotherapy Regime for BM	Concurrent systematic treatment	Brain metastasis (BM)	Changes of CSF mutation status						Response of BM	
									Pre-radiation		Post-radiation					
									Common driven mutation	Uncommon/rare/resistance mutation	Mutation abundance	Mutation number	New mutations	TMB	Best response	iPFS (days)
D01	Male	Squamous cell carcinoma CSF cytology (−)	2	1	Unknown	SIB-IMRT: WBRT 40 Gy/20F, GTV 56 Gy/20F	No	Newly diagnosed after CHE	No	No/No/PTEN, CDKN2A, ERBB2	↓	↓	No	↓	SD	> 662
D02	Female	Adenocarcinoma CSF cytology (−)	3	3	C-MET (+ + +) (Tissue from BM)	SIB-IMRT: WBRT 40 Gy/20F, GTV 56 Gy/20F	Pem + Cis	Newly diagnosed	No	No	–	–	–	–	SD	> 767
D03	Male	Adenocarcinoma CSF cytology (−)	2	2	EGFR 19-Del (Tissue from lung lesion)	SIB-IMRT: WBRT 36 Gy/20F, GTV 54 Gy/20F	Erlotinib	Newly diagnosed after TKI	No	No	↓	↓	EGFR 19del	↓	SD	> 675
D04	Male	Unknown CSF cytology (−)	3	1.5	Unknown	3D-CRT: WBRT 40 Gy/20F	No	Newly diagnosed	EGFR 19-Del	No	↓	↓	No	–	UN	219
D05	Female	Adenocarcinoma CSF cytology (−)	1	2.5	EGFR 19-Del (Tissue from lung lesion)	SIB-IMRT: WBRT 36 Gy/20F, GTV 56 Gy/20F	Erlotinib	Progressed after TKI	No	No	–	–	–	–	SD	385
D06	Male	Adenocarcinoma CSF cytology (+)	4	1.5	EGFR 21 L858R (+) (Tissue from lung lesion)	WBRT 40 Gy/20F SRS 16 Gy	Erlotinib	Progressed after TKI	EGFR 21 L858R (+)	No	↓ at first and then ↑	↑	CNVs	↑	UN	194
D07	Female	Adenocarcinoma CSF cytology (+)	4	0.5	EGFR 21 L858R (+) (Tissue from lung lesion)	3D-CRT: WBRT 30 Gy/10F	No	Newly diagnosed after CHE and TKI	EGFR 21 L858R (+)	No/No/MET amp, CDKN2A	↓	↓	MAP3K13 p.R585Q	No change	UN	20
D08	Female	Adenocarcinoma CSF cytology (−)	2	1.5	ALK(+) (Tissue from lung lesion)	SIB-IMRT: WBRT 40 Gy/20F, GTV 56 Gy/20F	Crizotinib	Newly diagnosed after TKI	ALK(+)	No/No/MDM2	slightly ↑	↓	No	↓	PR	> 600
S01	Female	Adenocarcinoma CSF cytology (+)	3	1	No (Tissue from lung lesion)	3D-CRT: WBRT 40 Gy/20F	Temozolomide + Osimertinib	Newly diagnosed after CHE	EGFR 19-Del	No/No/TP53, SMAD4	↑ at first and then slightly ↓	No change	No	No change	SD	> 414
S02	Female	Adenocarcinoma CSF cytology (+)	4	1	UN (Tissue from lung lesion)	3D-CRT:WBRT 36 Gy/18F	Osimertinib	Newly diagnosed after TKI	EGFR 21 L858R (+)	No/EGFR 21 A859S (+), EGFR 18 Q701L (+)/EGFR 20 T790M (+), PIK3CA MDM2	↓	No change	No	No change	UN	63
S03	Female	Adenocarcinoma CSF cytology (−)	3	1	EGFR 21 L858R (+) (Tissue from lung lesion + blood plasma)	SIB-IMRT: WBRT 36 Gy/20F, GTV 56 Gy/20F	Erlotinib	Newly diagnosed	EGFR 21 L858R (+)	No	↓	↓	PKHD1	↓	SD	274
S04	Male	Adenocarcinoma CSF cytology (−)	1	1.5	EGFR 18, 20 (+) (Tissue from lung lesion)	SIB-IMRT: WBRT 40 Gy/20F, GTV 56 Gy/20F	Pem + Cis	Progressed after CHE and TKI	No	No	↑	↑	EGFR 18 G719S	Slightly ↑	UN	–
S05	Female	Adenocarcinoma CSF cytology (−)	1	1.5	EGFR 21 L858R (+) (Tissue from left supraclavicular lymph node metastases)	SIB-IMRT: WBRT 40 Gy/20F, GTV 56 Gy/20F	Erlotinib	Newly diagnosed	EGFR 21 L858R (+)	No/No/MDM2	↓	↓	No	↓	SD	> 338
S06	Female	Adenocarcinoma CSF cytology (−)	3	1	EGFR 21 L858R (+) (Tissue from lung lesion)	SIB-IMRT: WBRT 40 Gy/20F, GTV 56 Gy/20F	Gefitinib	Progressed after TKI, CHE and Bev	EGFR 21 L858R (+)	No/No/TP53	↑ at first and then ↓	↑ at first and then ↓	SLC34A2	No change	PR	133
S07	Female	Adenocarcinoma CSF cytology (−)	3	1	EGFR 21 L858R (+) (Tissue from L4 vertebral metastases)	SIB-IMRT: WBRT 40 Gy/20F, GTV 56 Gy/20F	Erlotinib	Progressed after TKI	EGFR 21 L858R (+) MET amp	No/No/TP53, RB1	Slightly ↓	Slightly ↓	No	No change	SD	110
S08	Male	Adenocarcinoma CSF cytology (+)	4	0.5	EGFR 21 L858R (+) (Cerebrospinal fluid)	3D-CRT: WBRT 40 Gy/20F	Erlotinib + Bevacizumab	Newly diagnosed	EGFR 21 L858R (+)	No/No/TP53	↑	slightly ↑	RB1	No change	UN	41
S09	Male	Large cell lung cancer CSF cytology (−)	1	1	MET amplification (Tissue from lung lesion)	SIB-IMRT: WBRT 40 Gy/20F, GTV 56 Gy/20F	Crizotinib	Newly diagnosed	No	No	–	–	–	–	UN	61
S10	Male	Adenocarcinoma CSF cytology (−)	1	0.5	No (Tissue from lung lesion)	SIB-IMRT: WBRT 40 Gy/20F, GTV 56 Gy/20F	Pem + Cis	Progressed after SBRT	No	No	↓	↓	No	↓	PR	> 163
S11	Male	Adenocarcinoma CSF cytology (–)	1	1.5	EGFR exon 19 (+) (Tissue from right supraclavicular lymph node metastases)	SIB-IMRT: WBRT 40 Gy/20F, GTV 56 Gy/20F	No	Newly diagnosed after TKI	EGFR 19-Del	No/No/MYC, PIK3CA	↓	↓	No	↓	PR	> 148

### Influence of RT on cfDNA in CSF from NSCLC-BM patients

**Fig. 1:**
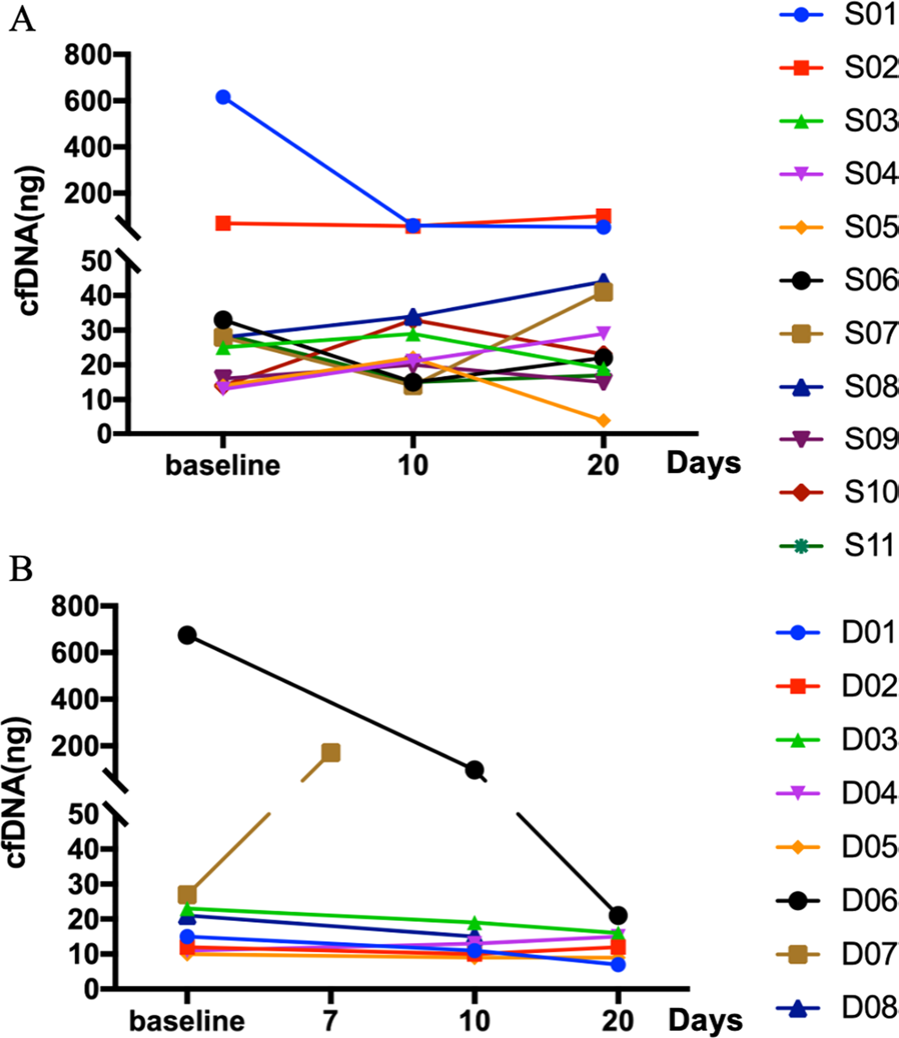
The amount of CSF cfDNA. **A** CSF cfDNA of patients D01-D08 collected at 0 day (baseline), 7th day and 10th day (during RT therapy), 20th day (after RT therapy) since the RT therapy began. **B** CSF cfDNA of patients S01–S11 collected at 0 day (baseline), 10th day (during RT therapy), 20th day (after RT) since the RT therapy began. *RT* radiation therapy

**Fig. 2:**
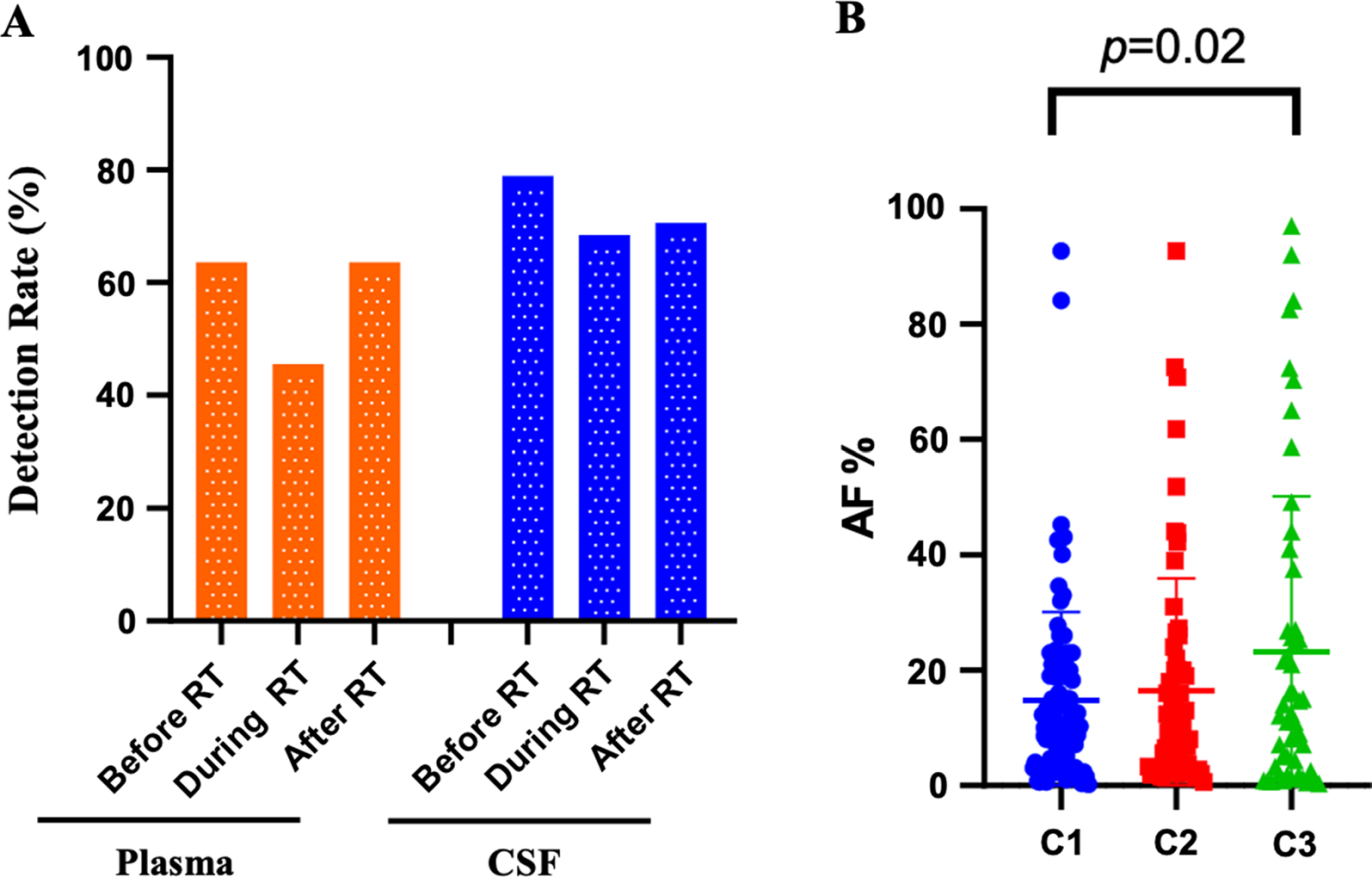
**A** The cfDNA detection rate of CSF and plasma. *RT* radiation therapy, *CSF* cerebrospinal fluid. **B** Genomic mutations abundance from the CSF. **C1** before RT, **C2** during RT, and **C3** after RT. *RT* radiation therapy, *CSF* cerebrospinal fluid

We compared cfDNA levels in the CSF before, during, and after radiotherapy. The amount of CSF cfDNA noticeable increased in D07 after RT, while the amount of CSF cfDNA decreased in D06 and S01, and no apparent change in the amount of CSF cfDNA occurred after RT in other patients (Fig. 1). And the detection rate of CSF cfDNA during RT and at the end of RT was slightly lower than before RT in the cohort. As expected, the detection rate of cfDNA was higher in CSF compared to plasma in the validation cohort (Fig. 2A). The composition of variants types was similar before and after RT in most patients except D06, with many copy number variants occurring at the end of RT (Additional file 1: Figure S2). A statistical increase in CSF mutation abundance was detected after RT compared to that before RT (Fig. 2B).

**Fig. 3:**
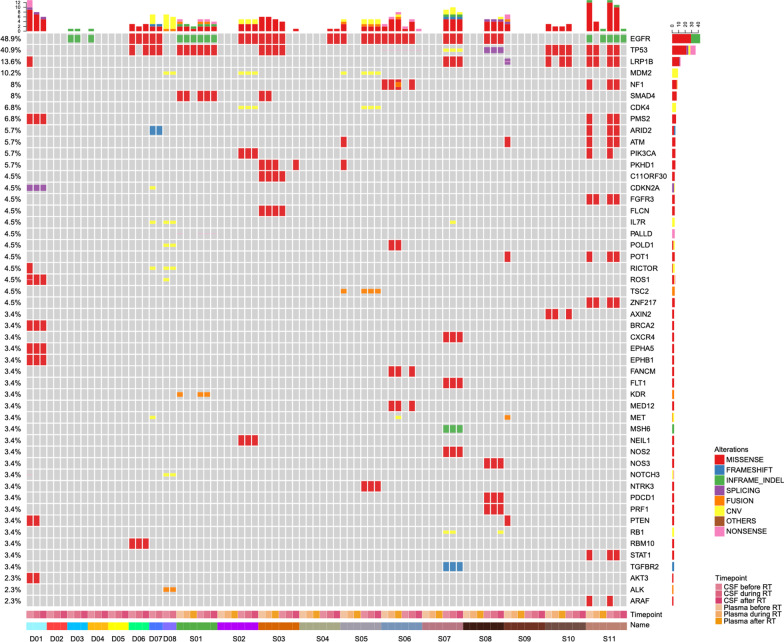
Genomic alterations in the CSF of training cohort at different timepoint. *INDEL* insertion-deletion, *CNV* copy number variation, *CSF* Cerebrospinal Fluid, *RT* radiation therapy

The trend is that mutation abundance in CSF increased in the beginning and then decreased subsequently during RT was observed in 13 patients (D01, D03, D04, D07, D08, S01, S02, S03, S05, S06, S07, S10, S11) while the numbers of mutations were stable or decreased in those patients (Fig. 3). Conversely, CSF mutations abundance and number of mutations obviously increased after RT occurred in D06, S04, and S08. Novel mutations were detected in 7 patients (D03, D06, D07, S03, S04, S06, S08) at the end of RT. And novel mutations detected in D03, S03, and S04 can also be detected in the paired tumor tissue or plasma samples. Additionally, no mutations were detected in the CSF before, during, or after RT in D02, D05, and S09 patients.

Compared to patients with BM who progressed after systematic treatment (2/5) or newly diagnosed patients with BM (2/6), rare, uncommon, or resistant mutations reported in previous studies were prone to be detected in patients with BM newly diagnosed after systematic treatment (6/7). And the number and abundance of those mutations decreased during RT.

**Fig. 4:**
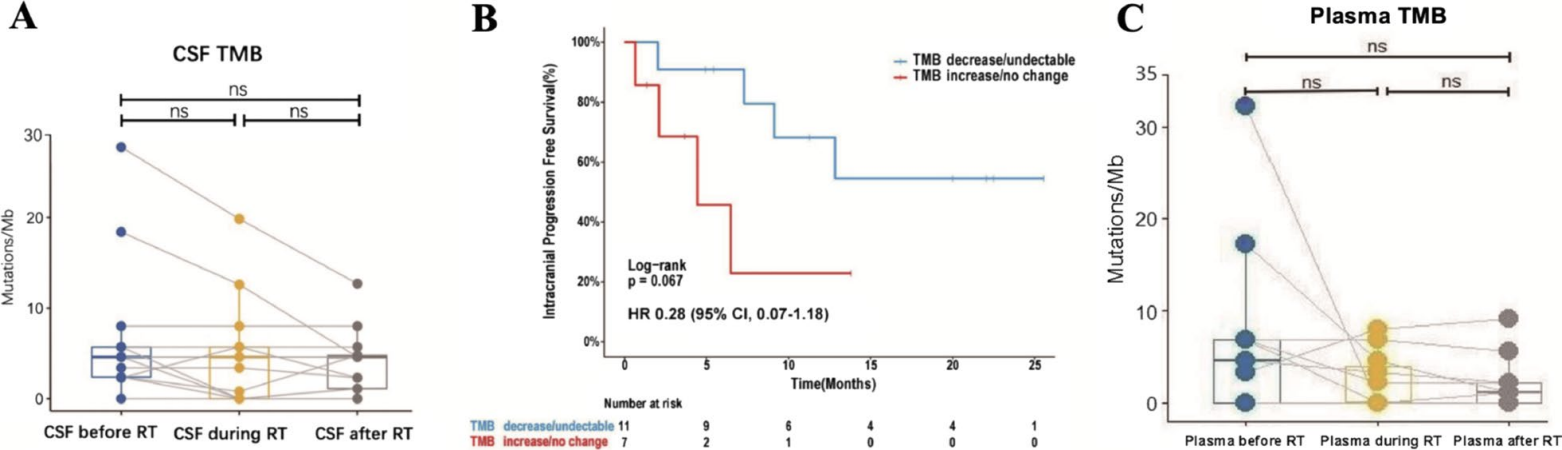
**A** Cerebrospinal fluid TMB change of cohort before RT, during RT, and after RT. No significant difference was observed. *CSF* cerebrospinal fluid, *TMB* tumor mutation burden, *RT* radiation therapy, *ns:* no significance. **B** Kaplan–Meier curves of intracranial progression free survival (iPFS) comparing cerebrospinal fluid TMB decrease/undetectable and TMB increase/no change. **C** Plasma TMB change before RT, during RT, and after RT. No significant difference was observed. *TMB* tumor mutation burden, *RT* radiation therapy, *ns* no significance

No significant difference was observed in CSF TMB (cTMB) before and after RT (Fig. 4A). Although the median iPFS of patients with decreased or undetectable cTMB has not been reached yet, there was a trend that these patients possessed longer iPFS compared to those with stable or increased cTMB (median: 4.43 months, range: 0.23–8.63 months) (HR 0.28, 95% CI 0.07–1.18, *P* = 0.067) (Fig. 4B).

### Risk factors of intracranial progression-free survival (iPFS)

To figure out independent risk factors predicting iPFS of patients undergoing RT for BMs with or without EGFR-TKIs, we further conducted subgroup analysis based on the diagnostic type of brain metastasis, leptomeninges involvement, performance status (PS) score, Graded Prognostic Assessment (GPA) score and detection of novel mutations at the end of RT (Additional file 1: Figure S3). However, no significant independent risk factors were found, probably due to the limited sample size and short follow-up time. However, in this study, the median iPFS of patients with BMs progression after initial EGFR-TKIs with or without chemotherapy (6.47 months) tends to be the shortest compared to patients with newly diagnosed BMs (not yet reached) and patients with newly diagnosed BMs after systematic treatment (with or without TKI) (9.13 months). Probable explanations for this phenomenon include those patients with BMs progression after initial EGFR-TKIs with or without chemotherapy substantially possessing dismal survival outcomes, or non-salvage RT could benefit patients with BMs. The exact reasons are worth further exploration.

### Influence of RT for BMs on blood immune indexes

Concordant with previous results, there was no significant difference in plasma TMB before and after RT (Fig. 4C). In addition, analyzing the change of T cell subsets in peripheral blood, we observed that the proportion of CD4^+^ naïve T cells significantly decreased after RT. Although, the change in the proportion of total T cells, natural killer (NK) cells and B cells is not statistically significant, there is an increasing trend of the proportion of total T cells and B cells during radiotherapy. The regulatory T (Treg) cells, a kind of as suppressor T cells, has no significant changes. We did not find significant changes in the proportion of other T cell subsets, including memory T cells (CD4^+^CD45RO^+^), cytotoxic T cells (CD3^+^CD8^+^), helper T cells (CD3^+^CD4^+^), CD8^+^CD28^+^ T cells, and CD8^+^CD28^−^ T cells (Additional file 1: Figure S4).

## Discussion

BM is a challenge for most clinicians, and whole brain radiotherapy (WBRT) is the acknowledged standard approach with proven efficacy for those patients. Although some studies on targeted therapies posed a challenge to RT in patients with driver gene mutation BM, a head-to-head clinical trial comparing efficacy between targeted therapy alone and combinations of targeted therapy with RT is still lacking. Thus, combining targeted therapy with RT for BMs is still a controversial question drawing intense clinical attention. Besides radiographic images, evaluation of the efficacy of RT alone or in combination therapy for BM from NSCLC remains limited. With the development of NGS, it provides a novel approach for evaluation of therapeutic efficacy by analysis of CSF.

A study presented at the American society of clinical oncology (ASCO) annual meeting 2020 reported that the median time from the initiation of RT (chest: 2.5–4.0 Gy/F; brain: 6–9 Gy/F) to the peak of the amount of ctDNA was two days (IQR:1–3 days) [21]. In plasma, ctDNA accounts for a small fraction of cfDNA because most cfDNA is derived from non-tumor cells, especially blood cells [22]. Compared with plasma cfDNA, most CSF cfDNA with high sensitivity is derived from tumor lysis. During and after radiotherapy, tumor cell destruction leads to the release of intracellular content including cfDNA. In the present study, the amount of CSF cfDNA significantly increased in only 5.3% (1/19) of patients. At the same time, 10.5% (2/19) of patients significantly reduced. There is no dynamic change in CSF cfDNA found during RT (10–14 days after initiation) and/or at the end of RT (26–28 days after initiation) compared to that before RT. Consistent rate of novel mutation detected in CSF cfDNA reported in previous studies, novel mutations were detected in CSF cfDNA after treatment in 21% (4/19) of patients in the present study. Notably, novel mutations detected in 3 patients (D03, S03, S04) CSF cfDNA at the end of RT, which were absent in CSF cfDNA before treatment, were also detected in corresponding lung tumor or plasma samples. It is suggested that radiation-induced tumor lysis might increase the sensitivity of the detection of CSF cfDNA.

It’s reported that the mechanism of acquired resistance to epidermal growth factor receptor tyrosine kinase inhibitors (EGFR-TKIs) includes *EGFR T790M* mutation, *MET* or *ERBB2* amplification, and a shortened time to progression of patients undergoing EGFR-TKIs was correlated with mutations in *ERBB2*, *MET,* and *TP53* [23]. Besides, the co-occurrence of deletion of *CDKN2A* and mutations of *EGFR* generally indicated poor treatment response to EGFR-TKIs in lung cancer [24, 25]. Subclones resistant to EGFR-TKIs might originate from various genetic alterations, including *TP53* mutation, *CDKN2A* deletion, and *RB1* deletion in tumor stem cells [25, 26]. Kenji Sugio reported that loss of *PTEN* might contribute to the acquired resistance of EGFR-TKIs in patients with uncommon *EGFR G719X* mutation [27]. *MDM2* amplification was found to induce the primary resistance of EGFR-TKIs and predict poor prognosis in NSCLC patients [28]. It’s reported that *CDC73*, *SMAD4*, *RB1,* and *PIK3CA* might also take an essential part in primary resistance to EGFR-TKIs [29]. Additionally, Keunchil Park found that genetic alterations of *TP53*, *RB1*, *PTEN,* and *MDM2* were also independently associated with worse PFS in patients taking third-generation TKI after initial EGFR-TKI failure [30]. Our study previously reported that mutations related to acquired or primary resistance to EGFR-TKIs were detected in 52.63% (10/19) of patients. Except for S08, the abundance or amount of EGFR-TKIs resistance-related mutations was decreased variously during RT in patients harboring these mutations. Notably, 70% (7/10) of these patients possessed longer iPFS (> 110 days), which indicates that RT alone or a combination of EGFR-TKIs with RT might effectively reduce or eliminate EGFR-TKIs resistant clones in BMs from NSCLC.

Considering the large gene panel (> 300 genes) used in the present study, we also calculated the CSF tumor mutation burden (cTMB) and explored its clinical value in patients with BMs. Notably, the median iPFS of patients with decreased or undetectable cTMB was longer than those without. However, this finding needs to be validated in multicenter, large cohorts. Moreover, whether cTMB can serve as a prognostic biomarker just like blood TMB remains largely unknown, and further research is needed. In addition, whether RT for BMs would stimulate the response of the immune system was also explored preliminarily in our study. Based on analyses of lymphocyte subsets in blood and plasma TMB, we found that the influence imposed on the immune system by RT for BMs was indeterminate.

## Conclusion

RT was associated with treatment benefits in advanced NSCLC patients with brain metastases. cfDNA in cerebrospinal fluid is more sensitive to the detection of mutated genes than that in plasma, which could be a more valuable predictive marker of radiotherapy efficacy. Although there was no significant difference in plasma TMB before and after RT, the proportion of CD4 + naïve T cells significantly decreased after RT. Taken together, these results indicate that radiation-induced tumor lysis might increase the sensitivity of the detection of CSF cfDNA. Besides, RT alone or a combination of EGFR-TKIs with RT might effectively reduce or eliminate EGFR-TKIs resistant clones in BMs from NSCLC. Evaluating a real-world population of NSCLC patients with BM will prove the predictive value of cfDNA in cerebrospinal fluid definitively in the future.

## Supplementary Information

**Additional file 1**. Supplementary figures.

**Additional file 2**. Supplementary tables.

## Data Availability

Datasets used and/or analyzed during the current study are available from the corresponding author on reasonable request.
