# Supplementary material for: Prognostic value of cell-free DNA in cerebrospinal fluid from lung cancer patients with brain metastases during radiotherapy
Source: Radiat Oncol. 2023 Mar 11;18:50. doi: 10.1186/s13014-023-02239-y (PMC10007729; doi:10.1186/s13014-023-02239-y)
Supplement: Supplementary file 1 — Additional file 1. Supplementary figures. [file 13014_2023_2239_MOESM1_ESM.docx]

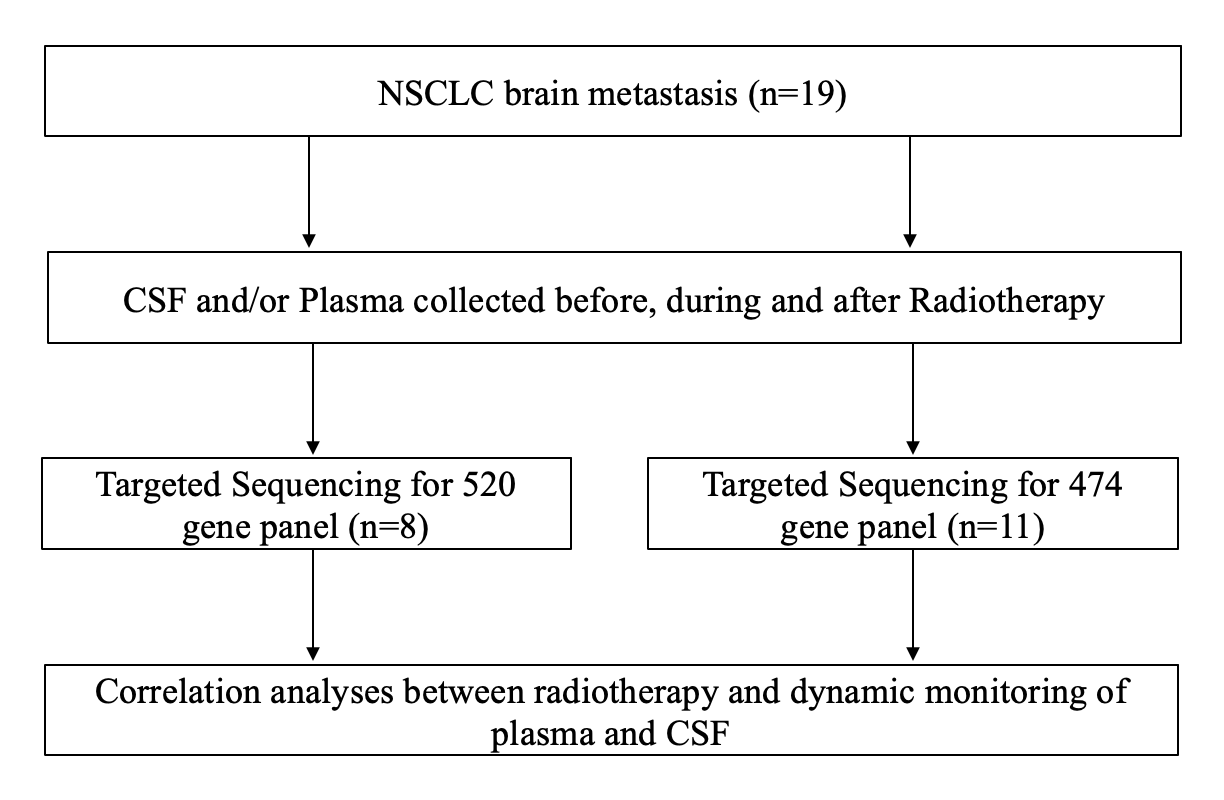


**Figure S1. Patients and study design. CSF cerebrospinal fluid.**

**
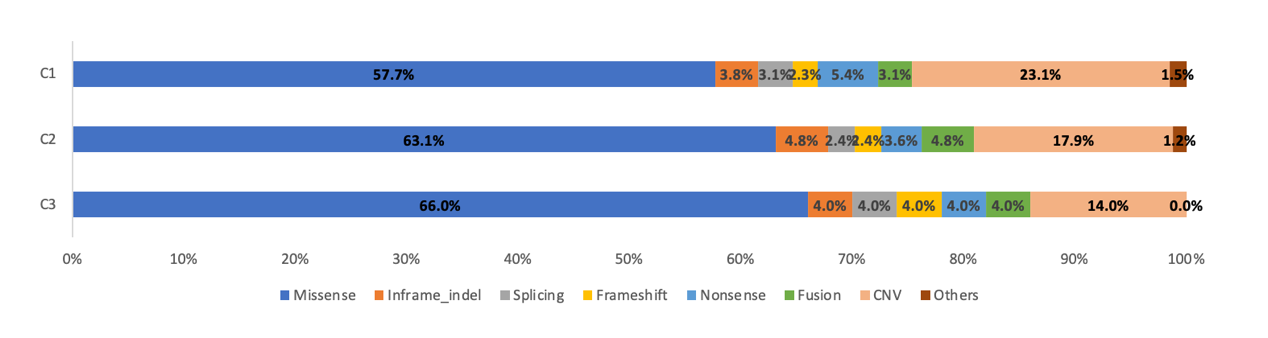
**

**Figure S2 Genomic variation types of CSF (C1) before RT, (C2) during RT and (C3) after RT. RT radiation therapy, CSF cerebrospinal fluid.**

**
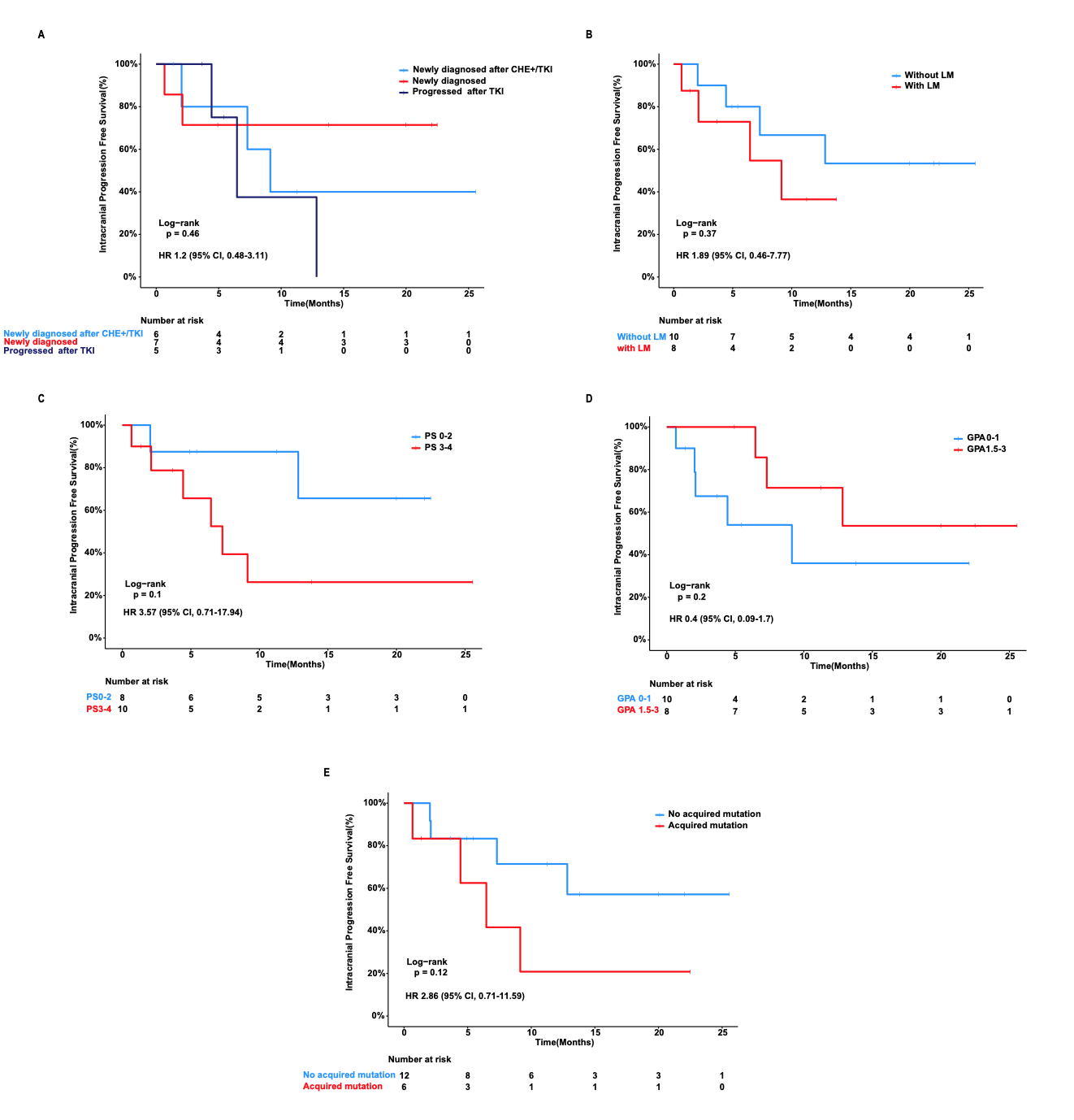
**

**Figure S3 Risk factors analyzed by Kaplan-Meier curves of intactanial progression freesurvival (iPFS).** (A) Diagnostic type: newly diagnosed after chemohearpy or TKI, newly diagnosed without previous treatment, and progressed after TKI. (B) Leptomeningeal metastases (LM) and no leptomeningeal metastases (C) performance status (PS) score 0-2 and 3-4，(D) Graded Prognostic Assessment (GPA) score 0-1 and 1.5-3.（E）Novel mutations detected and no novel mutation detected at the end of RT. RT radiation therapy.


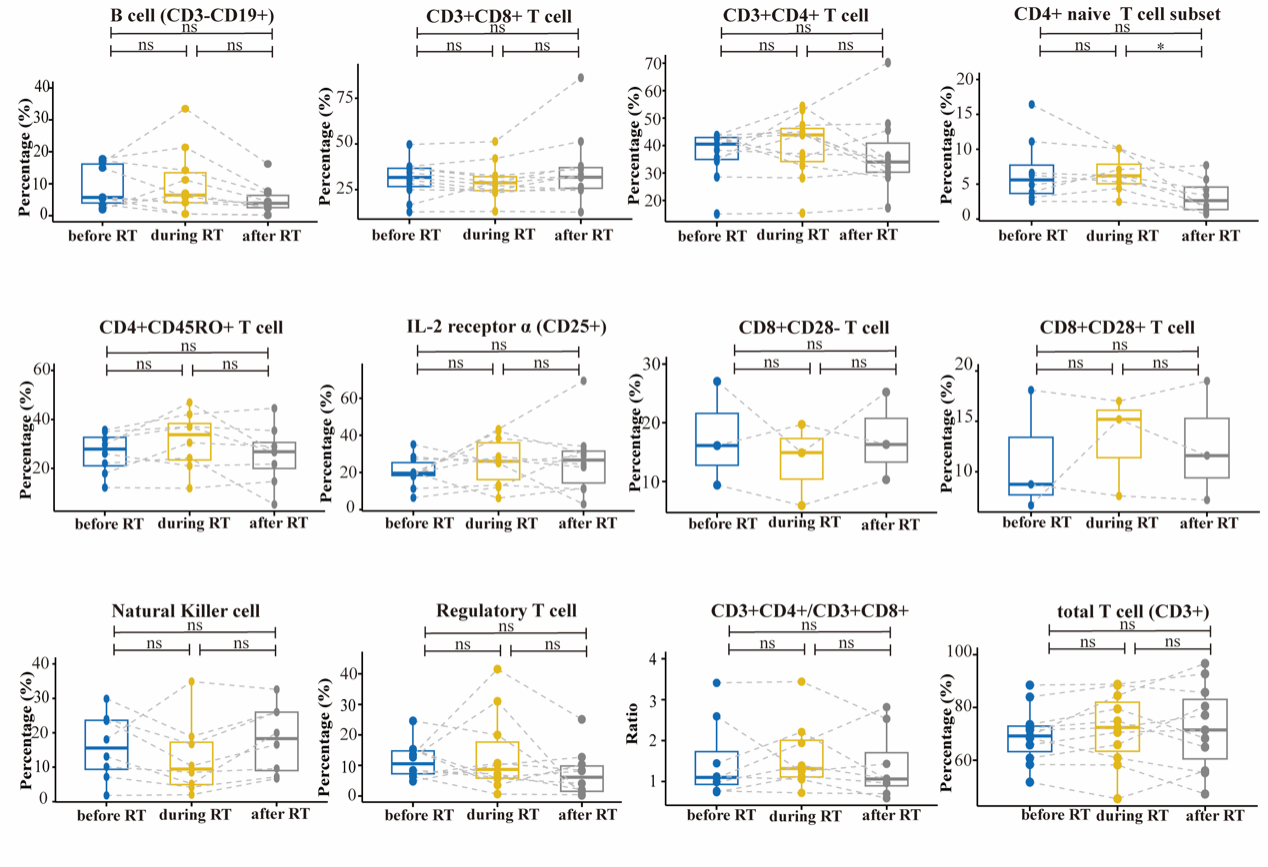


**Figure S4. T cell subsets analysis in peripheral blood before RT, during RT, and after RT. RT radiation therapy.**
