# Supplementary material for: Prognostic value of cell-free DNA in cerebrospinal fluid from lung cancer patients with brain metastases during radiotherapy
Source: Radiat Oncol. 2023 Mar 11;18:50. doi: 10.1186/s13014-023-02239-y (PMC10007729; doi:10.1186/s13014-023-02239-y)
Supplement: Supplementary file 2 — Additional file 2. Supplementary tables. [file 13014_2023_2239_MOESM2_ESM.docx]

**Table S1** Patient Characteristics

| **Item** | **Cohort (n=19)** |
| --- | --- |
|  |  |
|  |  |
| Gender |  |
| Male: Female | 9：10 |
| Age, y | 57（26-73） |
| Smoking history |  |
| Former/ Current | 4 |
| Never | 15 |
| Histology |  |
| Adenocarcinoma | 16 |
| Squamous carcinomas | 1 |
| Others/Unknown | 2 |
| Intracranial metastasis |  |
| Brain metastasis | 11 |
| Meningeal metastasis | 8 |
| Diagnosis type of BM |  |
| Newly diagnosed without previous treatment | 6 |
| Newly diagnosed after systemic treatment | 7 |
| Progression after systemic treatment | 5 |
| Progression after local treatment | 1 |
| PS score |  |
| 0-2 | 9 |
| 3-4 | 10 |
| GPA score |  |
| 0-1 | 10 |
| 1.5-3 | 9 |
| Baseline mutation status |  |
| EGFR 19del | 4 |
| EGFR 21（L858R） | 7 |
| EGFR rare mutation | 1 |
| ALK fusion | 1 |
| MET overexpression/ amplification | 2 |
| Unknown/ none | 4 |
| Synchronous system therapy |  |
| Gifitinib/ Erlotinib | 8 |
| Osimertinib | 2 |
| Crizotinib | 2 |
| Chemotherapy | 3 |
| None | 4 |
